# Determination of tissue oxygen saturation by diffuse reflectance spectroscopy

**DOI:** 10.1117/1.JBO.28.9.095002

**Published:** 2023-09-28

**Authors:** Laura Lucía Sánchez-Ramos, Beatriz Morales-Cruzado, Francisco Gerardo Pérez-Gutiérrez

**Affiliations:** a Universidad Autónoma de San Luis Potosí, Facultad de Ingeniería, San Luis Potosí, México; b CONACYT-Universidad Autónoma de San Luis Potosí, Facultad de Ingeniería, San Luis Potosí, México

**Keywords:** tissue oxygenation, diffuse reflectance, oxygen saturation, vascular occlusion

## Abstract

**Significance:**

Tissue oxygenation is a parameter that allows for determining the health status of human beings. In diabetic patients, it is particularly important to evaluate this parameter as an indicator of microcirculatory problems in the extremities.

**Aim:**

We aim to obtain tissue oxygen saturation from diffuse reflectance measurements.

**Approach:**

A computational algorithm to automate the methodology was implemented with the aim of establishing a medical diagnosis technique that is non-invasive and easy to apply and requires a short evaluation time. Tissue oxygen saturation measurements were performed on a group of volunteers to whom a vascular occlusion was applied. It was observed that, by increasing the applied pressure to the arm of each volunteer, the tissue oxygen saturation progressively decreased.

**Results:**

The results indicate that the developed technique is an effective method for monitoring changes in blood hemodynamics in patients with some type of pathology in which tissue oxygenation is compromised. In addition, the expected behavior of tissue oxygen saturation during a vascular occlusion was obtained.

**Conclusions:**

A methodology to obtain tissue oxygen saturation from diffuse reflectance measurements was successfully developed. It meets the necessary characteristics to be considered a technique for obtaining ${\mathrm{StO}}_{2}$ because it can be applied *in vivo* and non-invasively and does not require a high computational cost; thus it is fast and capable of providing an objective and quantifiable evaluation.

## 1 Introduction

Diabetic foot syndrome is one of the main complications that frequently affect the quality of life of people with diabetes mellitus. This syndrome includes a set of signs and symptoms linked to various vascular, neurological, and infectious disorders. Among the detection methods for an early diagnosis of diabetic foot, which have the objective of taking preventive actions, is the podiatric evaluation that consists of a subjective test known as the Semmes Weinstein monofilament, which evaluates diabetic peripheral neuropathy.^1^ There is also the ankle brachial index test, which is used to compare blood flow in the arteries of the arm and ankle and represents an indicator of vascular disrepair.^2^ Another method used to evaluate vascular deterioration is Doppler ultrasound, which uses a transducer to emit sound waves that bounce off blood vessels to create images of the state of those vessels.^3^ Although these methods can provide important information about the condition of patients with diabetic foot, they generally require a long test time and, in some cases, these do not provide a quantitative result of the analyzed condition.

Optical technologies have emerged as promising tools for medical diagnosis with several advantages such as non-ionizing radiation, low cost, and high molecular and biochemical specificity.^4^ One of the main applications of optical technologies is oximetry, which is used to measure the oxygenation level of the blood through optical measurements of the transmission/reflection of light through the blood, which is based on the different absorption spectra of oxygenated (${\mathrm{HbO}}_{2}$) and deoxygenated (Hb) hemoglobin.

Spectroscopy is one of the techniques used for the evaluation of blood microcirculation because it allows for the analysis of the optical properties of the tissue. Because biological molecules have unique absorption spectra as a function of the wavelength of light, it is possible to detect a precise concentration of these molecules by spectroscopy. Research applying various optical spectroscopy techniques, mainly in the advanced stages of diabetic foot, has been conducted this includes hyperspectral imaging,^5^ diffuse photon density wave,^6^ frequency-domain diffuse near-infrared spectroscopy,^7^ non-contact near-infrared imaging system,^8^ and terahertz reflection.^9^ Although most studies focus on the implementation of near infrared spectroscopy technology, there are certain disadvantages such as the positioning of the optical probes and the separation between the emitter and receiver as this separation distance influences the penetration depth of the light beam. Therefore, a technique that provides an objective and quantifiable evaluation is required.

Because blood microcirculation is affected in patients with diabetic foot syndrome, tissue oxygenation is one of the most relevant parameters during the disease process. Because foot ulcers occur when the lack of tissue oxygen prevents cell regeneration, non-invasive quantitative measurements of hemoglobin concentration and tissue oxygen saturation can provide information for diagnostic and therapeutic decision making. Currently, there is no optical technique that is easy to use, reliable, and economical to monitor tissue oxygenation, so this work proposes to cover these needs. The technique is based on diffuse reflectance spectroscopy, which is an especially interesting non-invasive optical technique because the spectrum acquired to apply this technique presents information about the chromophores (absorbent elements) of the tissue and its morphology. Because blood is the dominant light absorber in the visible wavelength range, both local blood content and tissue oxygen saturation can be extracted from the reflected light signal using an integrating sphere. The results of the work presented here demonstrate that it is feasible to implement a portable device to monitor tissue oxygen saturation in clinical practice. Such a device works with light emitting diode (LED) lighting and detection with photodiodes.

## 2 Methodology

### 2.1 Oxygen Saturation

Due to the critical nature of tissue oxygen consumption in the body, oxygen saturation, the amount of hemoglobin bound to oxygen compared with the amount of unbound hemoglobin, is an essential element of patient care; thus it is currently considered to be the fifth vital sign.^10^

Blood oxygenation can be evaluated by monitoring oxygenated hemoglobin (${\mathrm{HbO}}_{2}$) and deoxygenated or reduced hemoglobin (Hb) concentration parameters, which are also used to calculate the ${\mathrm{SO}}_{2}$ oxygen saturation parameter as

$${\mathrm{SO}}_{2}=\frac{\left[{\mathrm{HbO}}_{2}\right]}{\left[{\mathrm{HbO}}_{2}]+[\mathrm{Hb}\right]}x100\%,$$ (1)

where oxygen saturation ${\mathrm{SO}}_{2}$ represents the concentration of oxygenated hemoglobin in relation to total hemoglobin. ${\mathrm{SO}}_{2}$ takes different names depending on where it is measured. For example, ${\mathrm{SaO}}_{2}$ for artery, ${\mathrm{SvO}}_{2}$ for veins, and ${\mathrm{StO}}_{2}$ for tissue. Thus, ${\mathrm{StO}}_{2}$ is the ratio between oxygenated hemoglobin and total hemoglobin in all tissue blood: arterioles, capillaries, and venules. It is directly related to tissue blood flow and inversely related to its metabolic requirements.

Kyriacou et al.^11^ present the molar extinction coefficients or specific absorption constants of ${\mathrm{HbO}}_{2}$ and Hb as a function of wavelength, in the visible and near-infrared wavelength regions, where a large difference between the extinction coefficients of ${\mathrm{HbO}}_{2}$ and Hb is clear for some wavelengths. Another important aspect is that ${\mathrm{HbO}}_{2}$ and Hb have a different absorption throughout the spectrum, except at 805 nm, which is called the isosbestic point. For shorter wavelengths, the absorption is mainly due to Hb, whereas for longer wavelengths, most of the absorption is due to ${\mathrm{HbO}}_{2}$.

To obtain the oxygen saturation value using Eq. (1), it is necessary to select at least two wavelengths considering three main criteria: first, the selected wavelengths ($\lambda_{1}$ and $\lambda_{2}$) must be sensitive to each of the main blood absorbers, ${\mathrm{HbO}}_{2}$ and Hb (which are generally opposite the isosbestic point). Second, the absorption difference between ${\mathrm{HbO}}_{2}$ and Hb at the chosen wavelengths should be the largest. Finally, the absorption spectra in the region of the chosen wavelengths should be relatively flat, indicating their proximity in terms of absorption magnitude.^11^

### 2.2 Propagation of Optical Radiation in Human Skin

The interval of the electromagnetic spectrum in which the tissues have a significant level of light penetration to provide information on the state of the tissues, but without causing a harmful effect on health, is known as the therapeutic window and is located between the wavelengths of 600 and 1300 nm.^12^

The interactions of electromagnetic radiation with the skin are governed by its physiological, biochemical, and morphological characteristics.^13^ They are characterized by a strong optical scattering due to the high degree of inhomogeneity caused by the organelles that the cells contain, which have different sizes and compositions; likewise they present a different refractive index to that of the substance in which the fibers are immersed and connective tissue cells.^14^ Therefore, the skin can be considered to be an inhomogeneous turbid medium.

When electromagnetic radiation interacts with the skin, a part of the radiation is reflected due to the difference between the refractive indices of the skin and the air, and the remaining radiation that crosses the border can be absorbed internally or scattered multiple times by the middle. Radiation that has undergone multiple scattering events can be diffusely transmitted or reflected.^15^ To describe the propagation of electromagnetic radiation in the skin, the absorption coefficient ($\mu_{a}$), scattering coefficient ($\mu_{s}$), anisotropy factor (g), and refractive index (n) are used as the most important optical properties.^16^

Because human skin is a tissue made up of different layers that contain a diversity of non-homogeneous structures, the study of the interaction of electromagnetic radiation with this medium becomes difficult due to the diversity in the size, shape, density, and refractive index of these components. Therefore, simplifications are generally made for their study, and the structures are considered to be homogeneously distributed spherical particles.^17^

### 2.3 Radiative Transport Theory

The radiative transport theory considers that the only interaction of light with the medium is of an elastic type, changing the energy distribution of the incident light as it passes through such a medium.^18^^–^^21^ This theory does not include the effects of coherence, polarization, interference, and diffraction. Its fundamental equation is the radiative transfer equation (RTE), which is an integral–differential equation. The radiance or specific intensity is the unknown quantity in this equation and includes parameters such as the scattering and absorption coefficients and the phase function of the medium.

To solve the RTE, the main problem that arises is the evaluation of the diffuse radiance because the scattered photons do not follow a determined trajectory. For this reason, appropriate statistical approximations and approaches must be chosen, that is, whether absorption or scattering is the dominant attenuation process.

The methods used are generally the Kubelka–Munk theory, the diffusion approximation, the Monte Carlo method, or the adding–doubling method. Each method is based on certain approximations that facilitate the RTE solution. In general, the complexity of any of the approaches is closely related to its precision, as well as to the computation time required to give a solution.

The inverse adding–doubling (IAD) method provides a tool for the fast and accurate solution of the inverse scattering problem. Adding–doubling is based on the general method for the solution of the RTE for flat and parallel layers, suggested by Van de Hulst and introduced to the optics of biological tissues by Prahl et al.^22^

An important advantage of the IAD method when applied to biological tissue optics is the possibility of rapidly obtaining iterative solutions. Furthermore, it is flexible enough to consider scattering anisotropy and internal reflection from sample boundaries.

### 2.4 Experimental Set-Up

**Fig. 1:**
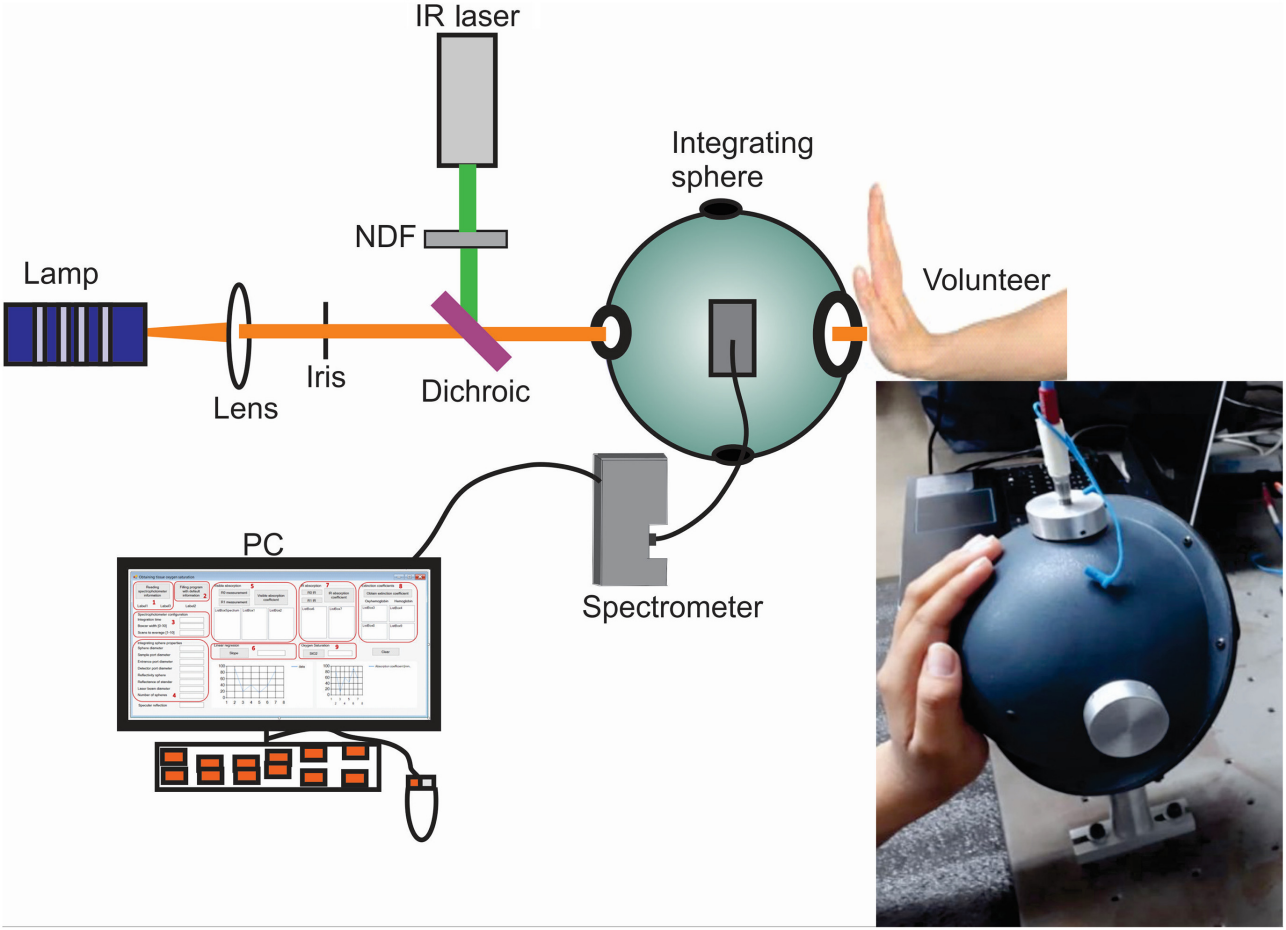
Experimental set-up for diffuse reflectance measurement. Inset: integrating sphere used to measure diffuse reflectance of the volunteer hand.

To obtain tissue oxygen saturation, it is necessary to obtain the concentrations corresponding to oxygenated (${\mathrm{HbO}}_{2}$) and deoxygenated (Hb) hemoglobin. Those concentrations can be determined from the absorption coefficients of the tissue to be analyzed. The absorption coefficients are recovered by IAD from diffuse reflectance measurements made with an integrating sphere experimental set-up (see Fig. 1). The experimental set-up was used to measure the reflected light by the sample; then it is normalized and used as input parameter together with the scattering coefficient and the anisotropy factor.^23^

The experimental set-up consists of a tungsten lamp (HL-2000; Ocean Optics) that emits radiation in the visible spectrum (even though the spectrum is specified from 360 to 1700 nm) coupled to an optical fiber that carries light from the lamp to the input port of an integrating sphere (819C-SF-6; Newport) passing through a collimating lens (fiber optics do not penetrate the integrating sphere). The collimated light reaches the sample, which is placed in the opposite port to the entrance of the light source and is reflected in a specular and diffusive way. An optical fiber connected to the top port of the integrating sphere carries the diffusively reflected light from the sample to a spectrometer (USB4000; Ocean Optics, with a detection range between 200 and 1100 nm). A continuous wave collimating laser (BWF-975-450E) with a central wavelength of 975 nm and a maximum output power of 450 mW was used as the light source. For the experiments, only 1.0 mW was used in an area of $2.27\;{\mathrm{mm}}^{2}$, which is harmless for human skin.

The reflectance measurement was performed by placing the palm of the hand on the outlet port of the integrating sphere, as shown in Fig. 1. The light reflected by the tissue is collected and guided to the spectrometer by means of an optical fiber, and the spectrometer separates the reflected light at different wavelengths and provides a spectrum of the intensity.

**Fig. 2:**
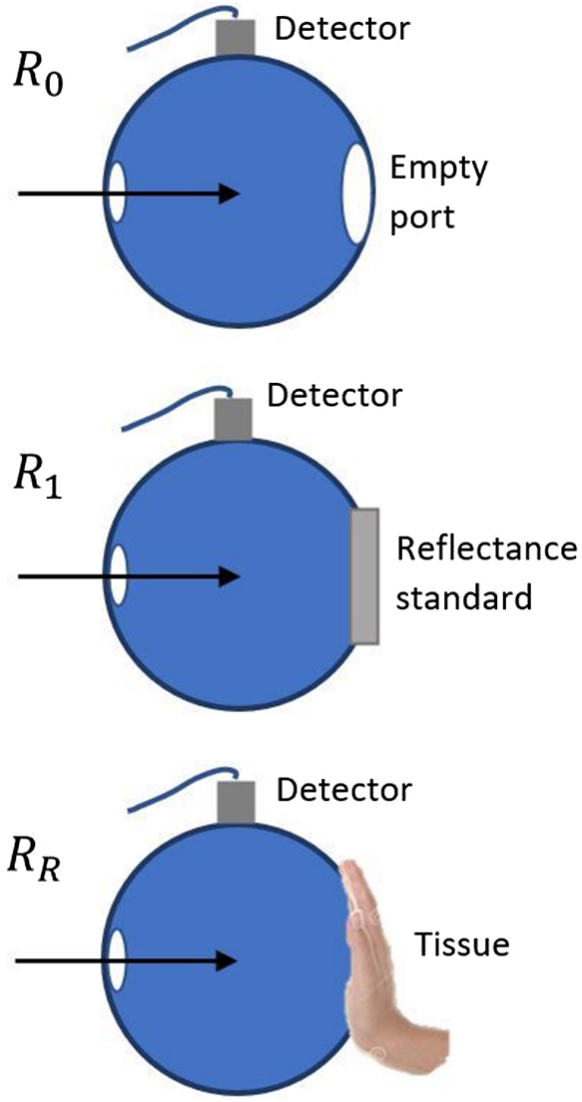
Schematic representations of the measurement of $R_{0}$, $R_{1}$, and $R_{R}$ for calculation the of $M_{R}$.

The reflection measurements are normalized with respect to the incident light. Applying Eq. (2), a value of the total diffuse reflectance is obtained as

$$M_{R}=r_{\mathrm{std}}\cdot \frac{R_{R}-R_{0}}{R_{1}-R_{0}}+R_{\text{Fresnel}},$$ (2)

where $r_{\mathrm{std}}$ represents the reflection factor of the integrating sphere and the reference standard, $R_{R}$ is the reflection measured from the palm of the volunteer’s hand, $R_{0}$ is equivalent to the minimum reflection that can be measured and represents the electronic noise that is measured leaving the output port empty, and $R_{1}$ is the maximum reflection that can be measured and corresponds to the spectrum of the lamp that is measured by placing the reflection standard on the output port. Measurements $R_{0}$, $R_{1}$, and $R_{R}$ are the result of averaging several spectrophotometer readings with an integration time of 100 ms and a boxcar width of 50. The combination of both factors, integration time and boxcar width, with the amount of light emitted by the sources provides a high signal to noise ratio. The term $R_{\text{Fresnel}}$ refers to the Fresnel or specular reflection produced by the difference between the refractive indices of air and skin. Figure 2 shows the schematic representations of the measurements of $R_{0}$, $R_{1}$, and $R_{R}$.

The experimental tests were performed on healthy volunteers; for this reason, it was necessary to induce ischemia to simulate the reduction in blood flow that occurs in diabetic patients with peripheral vascular disease. A vascular occlusion test was performed using a sphygmomanometer; the blood pressure cuff was placed around the upper left arm and inflated to stop the flow of blood in the arteries/veins up to a certain pressure value indicated on the manometer. The vascular occlusion test has been used in various studies such as those carried out by Wang et al.,^24^ who applied the occlusion test to healthy volunteers to evaluate their hybrid diffuse optical device in stroke diagnosis. Donati et al.^25^ used the occlusion test in 27 healthy volunteers to detect alterations in tissue oxygenation and microvascular reactivity with the aim of predicting adverse outcome in critically ill patients. Bezemer et al.^26^ applied the test to eight healthy volunteers to assess potential metabolic and microcirculatory alterations that patients may suffer during sepsis or shock.

**Fig. 3:**
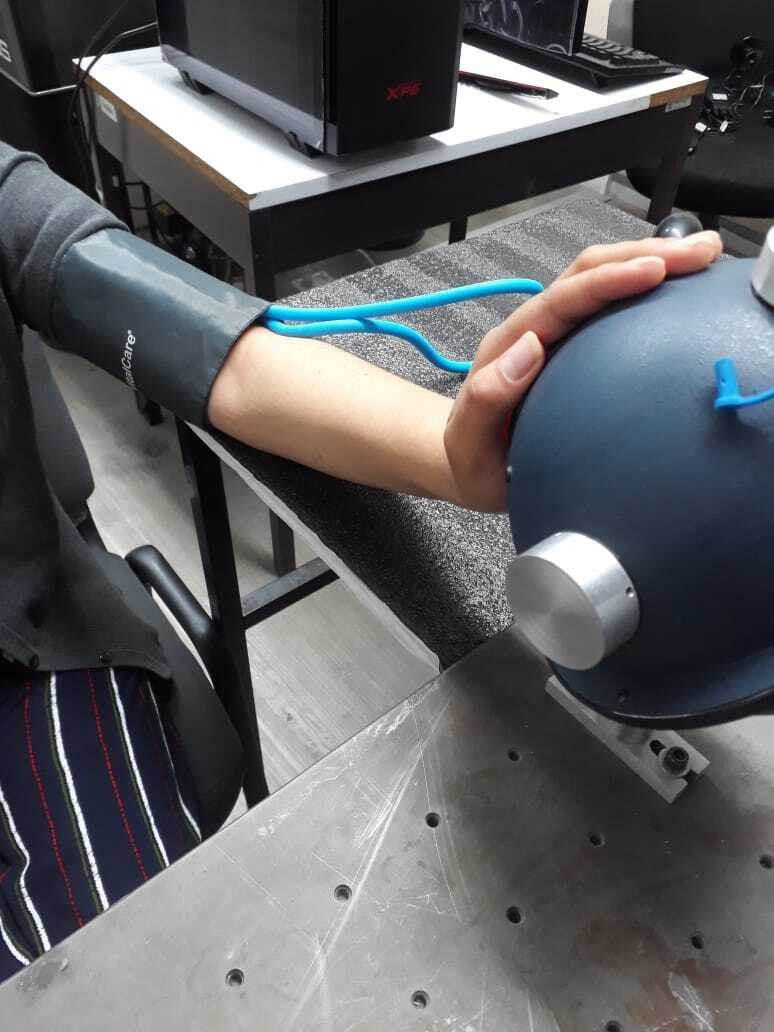
Setting of a sphygmomanometer for measurements of ${\mathrm{StO}}_{2}$.

The study followed the tenets of the Declaration of Helsinki and the Mexican General Health Law on Research for Health, and the corresponding protocol was approved by the Ethics and Research Committee CONBIOETICA of the UASLP (registration number CEI-FE-046-023). The volunteers were informed in detail about the experimental procedure, and they provided written consent for their data to be used for research purposes. The group of participants consisted of 27 healthy volunteers, 13 men and 14 women, between 18 and 39 years old. For the experimental tests, volunteers were asked to sit comfortably in a chair and rest for 3 min. During this time, blood pressure, heart rate, and pulsatile oxygen saturation were measured. In addition, a diffuse reflection spectrum was taken from each volunteer to calculate their absorption spectrum (see Supplementary Material). The absorption coefficient at 632 nm was correlated with melanin content, according to a prior study^27^ and classified into skin type on the Fitzpatrick scale,^28^ with the volunteers being between skin types III and V. Subsequently, a sphygmomanometer around the left arm was placed to induce vascular occlusions, and the palm of their left hand was placed on the port of the integrating sphere, as shown in Fig. 3.

Four continuous measurements were made at 0, 60, 80, and 100 mmHg of the 27 adult volunteers. Each occlusion took ∼1  min. At the end of the 100 mmHg measurement, the pressure in the cuff was released, and measurements were taken in the recovery period 1 and 2 min later. Volunteer characteristics such as age, blood pressure, heart rate, pulsatile oxygen saturation, and skin type were registered. The information of each volunteer was tagged using a consecutive number followed by their gender in parentheses.

### 2.5 Algorithm for Obtaining Oxygen Saturation

The tissue absorption coefficient $\mu_{a}$ can be considered to be the sum of n products of the molar extinction coefficients of the significant chromophores $\varepsilon_{i}$ and their respective concentrations $C_{i}$, where (λ) indicates a wavelength dependence, as calculated in Eq. (3):

$$\mu_{a}(\lambda )=\overset{n}{\underset{i=1}{\sum }}\varepsilon_{i}(\lambda )C_{i}.$$ (3)

From Eq. (2), concentrations of oxygenated hemoglobin ${\mathrm{HbO}}_{2}$ and deoxygenated or reduced hemoglobin Hb were obtained from the tissue absorption coefficients $\mu_{a}$ and molar extinction coefficients ε corresponding to each hemoglobin in two wavelengths $\lambda_{1}$ and $\lambda_{2}$, as indicated in Eqs. (4) and (5):

$$[{\mathrm{HbO}}_{2}]=\frac{\mu_{a}^{\lambda 2}\varepsilon_{\mathrm{Hb}}^{\lambda 1}-\mu_{a}^{\lambda 1}\varepsilon_{\mathrm{Hb}}^{\lambda 2}}{\varepsilon_{\mathrm{Hb}}^{\lambda 1}\varepsilon_{{\mathrm{HbO}}_{2}}^{\lambda 2}-\varepsilon_{\mathrm{Hb}}^{\lambda 2}\varepsilon_{{\mathrm{HbO}}_{2}}^{\lambda 1}},$$ (4)

$$[\mathrm{Hb}]=\frac{\mu_{a}^{\lambda 1}\varepsilon_{{\mathrm{HbO}}_{2}}^{\lambda 2}-\mu_{a}^{\lambda 2}\varepsilon_{{\mathrm{HbO}}_{2}}^{\lambda 1}}{\varepsilon_{\mathrm{Hb}}^{\lambda 1}\varepsilon_{{\mathrm{HbO}}_{2}}^{\lambda 2}-\varepsilon_{\mathrm{Hb}}^{\lambda 2}\varepsilon_{{\mathrm{HbO}}_{2}}^{\lambda 1}}.$$ (5)

where ${\mathrm{HbO}}_{2}$ and Hb have characteristic absorption spectra, reported on the Oregon Medical Laser Center website.^29^ These spectra correspond to the molar extinction coefficients of oxygenated $\varepsilon_{{\mathrm{HbO}}_{2}}$ and deoxygenated $\varepsilon_{\mathrm{Hb}}$ hemoglobin as a function of wavelength for the visible and near infrared regions, where the molar extinction coefficient is defined as an absorption constant per molar unit.

Due to the difference between $\varepsilon_{{\mathrm{HbO}}_{2}}$ and $\varepsilon_{\mathrm{Hb}}$ identified in the visible and near infrared region, these coefficients can be used for the evaluation of blood oxygenation.^30^ The chosen wavelengths to obtain the ${\mathrm{StO}}_{2}$ in this work were 975 nm in the infrared region, as well as a wavelength in the visible region positioned at a similar distance from the isosbestic point as the infrared point and showing significant changes in the ${\mathrm{StO}}_{2}$.

The absorption coefficients $\mu_{a}^{\lambda 1}$ and $\mu_{a}^{\lambda 2}$ in Eqs. (3) and (4) were obtained from the *in vivo* measurements of diffuse reflectance with the integrating sphere experimental set-up and following the procedure described by Quistian-Vázquez et al.^23^ to obtain the absorption coefficient $\mu_{a}$. The input parameters were the measurement of the diffuse reflectance $M_{R}$, the scattering coefficient $\mu_{s}$, and the anisotropy factor g of the skin; these depend on the biochemical composition of the tissue and its cellular structure, as well as on the wavelength.

The values of $\mu_{s}^{\prime }$ as a function of wavelength were obtained from Jacques’ review.^31^ Equation (6) characterizes scattering in seven tissue groups: skin, brain, breast, bone, soft tissues, fibrous tissues, and fatty tissues. The values of a and b for skin are $a=46{\;\mathrm{cm}}^{-1}$ and b=1.421, respectively,

$$\mu_{s}^{\prime }(\lambda )=a{\left(\frac{\lambda }{500\;\mathrm{nm}}\right)}^{-b}.$$ (6)

To define g(λ) as a function of the wavelength, a sixth order polynomial was determined; it represents a polynomial fit of the values obtained by Ma et al.^32^ in a study carried out to determine the optical properties of the skin and that are presented in the Jacques review.^31^ Equation (7) shows the function obtained for g(λ):

$$g(\lambda )=-5.603+3.61\times 10^{-2}\cdot \lambda -8.17\times 10^{-5}\cdot \lambda^{2}+9.51\times 10^{-8}\cdot \lambda^{3}\;-5.92\times 10^{-11}\cdot \lambda^{4}+1.83\times 10^{-14}\cdot \lambda^{5}-2.11\times 10^{-18}\cdot \lambda^{6}.$$ (7)

The scattering coefficient $\mu_{s}(\lambda )$ as a function of the wavelength was obtained from the relation $\mu_{s}^{\prime }=\mu_{s}(1-g)$.

## 3 Computer Program: Obtaining Tissue Oxygen Saturation

The algorithm “obtaining tissue oxygen saturation (OTOS)” was developed, taking as a reference the program developed by Quistian-Vázquez et al.,^23^ which uses a series of routines ranging from optical spectrometer reading until the retrieval of the absorption or scattering optical properties applying IAD. The Quistian-Vázquez program implemented *in vivo* measurements to monitor the absorption coefficient in real time. In a study carried out to evaluate the effect of temperature, results demonstrated a temperature dependence on the optical properties of human skin, which is important in the prescription of light doses based on optical properties. These results indicate the feasibility of using the integrating sphere in measurements of optical properties *in vivo* to monitor the state or health condition of people. On the other hand, Rehman et al.^33^ stated that the integrating sphere system together with the IAD method can differentiate between normal and diseased skin tissue based on the contrast in scattering and absorption properties.

To obtain the absorption coefficient $\mu_{a}$, the measurement of diffuse reflectance is required as input parameter $M_{R}$ and the optical properties of human skin as scattering coefficient $\mu_{s}$ and anisotropy factor g. The values of $\mu_{s}^{\prime }$ and g as a function of wavelength were obtained from Eqs. (6) and (7), respectively. Subsequently, the wavelength range from 600 to 700 nm was defined to retrieve the absorption coefficient in the visible region of the spectrum and to determine a linear fit in the range of 630 to 700 nm. This slope is related to the melanin content present in the skin, as demonstrated by Kollias and Baqer^34^ and Stamatas et al.^35^ Routines for reading the molar extinction coefficients of oxygenated and deoxygenated hemoglobin and to calculate tissue oxygen saturation were also included in the computer program. The values of $\varepsilon_{{\mathrm{HbO}}_{2}}$ and $\varepsilon_{\mathrm{Hb}}$ considered in this work are those reported by Prahl.^29^

**Fig. 4:**
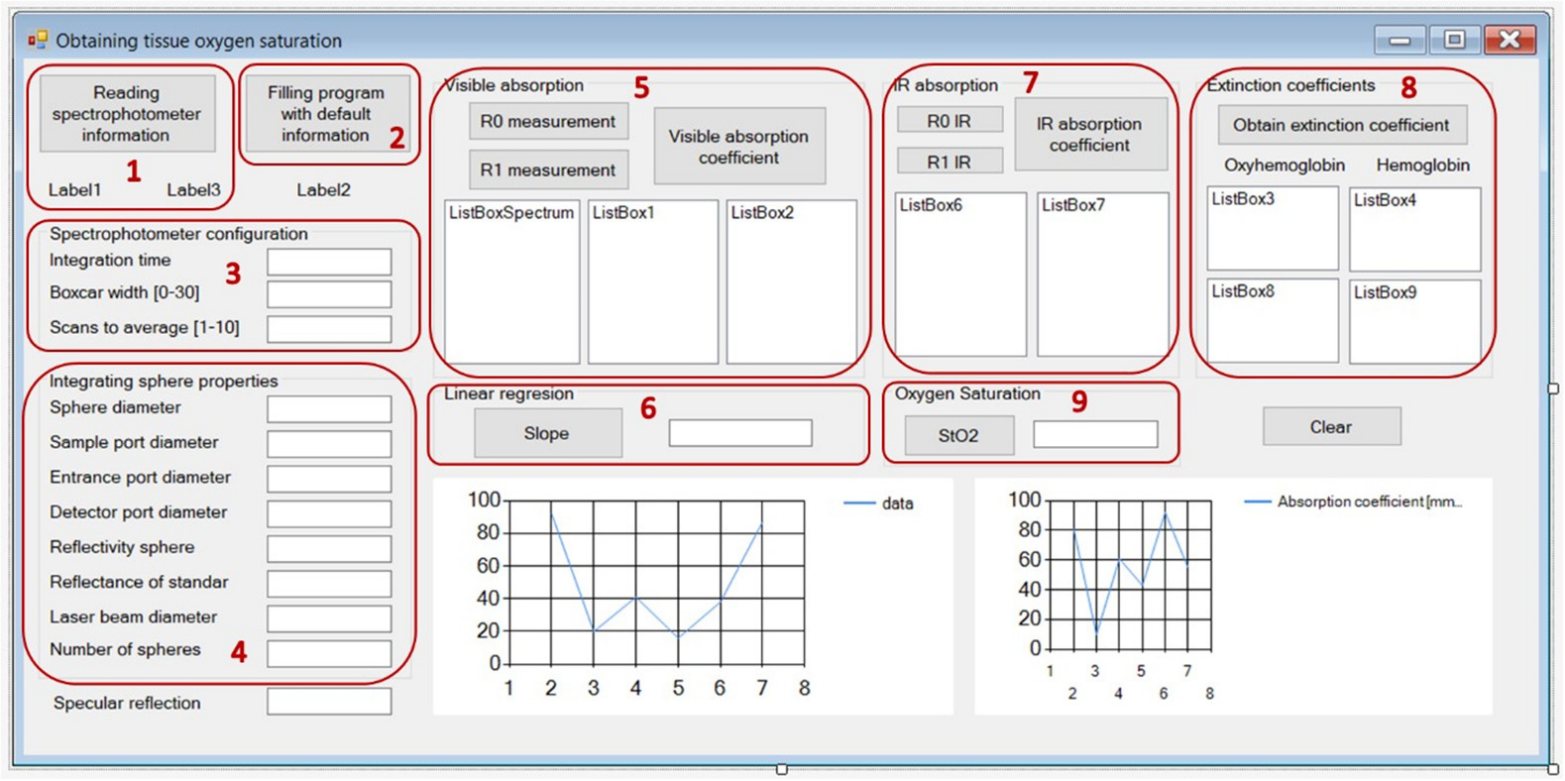
Parts of the graphical interface corresponding to the different stages of the algorithm for OTOS.

The graphical interface, as shown in Fig. 4, allows the user to define the input values for the spectrometer configuration (parts 1–3), as well as the properties of the integrating sphere (part 4). It also allows for viewing the values of the absorption/extinction coefficients and tissue oxygen saturation, both in the visible region and in the infrared point, as observed in parts 5–9.

The implemented algorithm to obtain tissue oxygen saturation allows for automating the measurements because it optimizes the processing time between each measurement. The implementation of the graphical interface facilitates the application of the algorithm by anyone because it does not require specific technical training. In this way, the developed algorithm provides a good tool for use in clinical practice, such as is routine in appointments for the control and follow-up of diabetic patients, for which medical diagnostic techniques that take up less evaluation time, are not invasive, and are easy to apply are required.

## 4 Results and Discussion

### 4.1 Measurement of Absorption Coefficient Spectra *In Vivo*

**Fig. 5:**
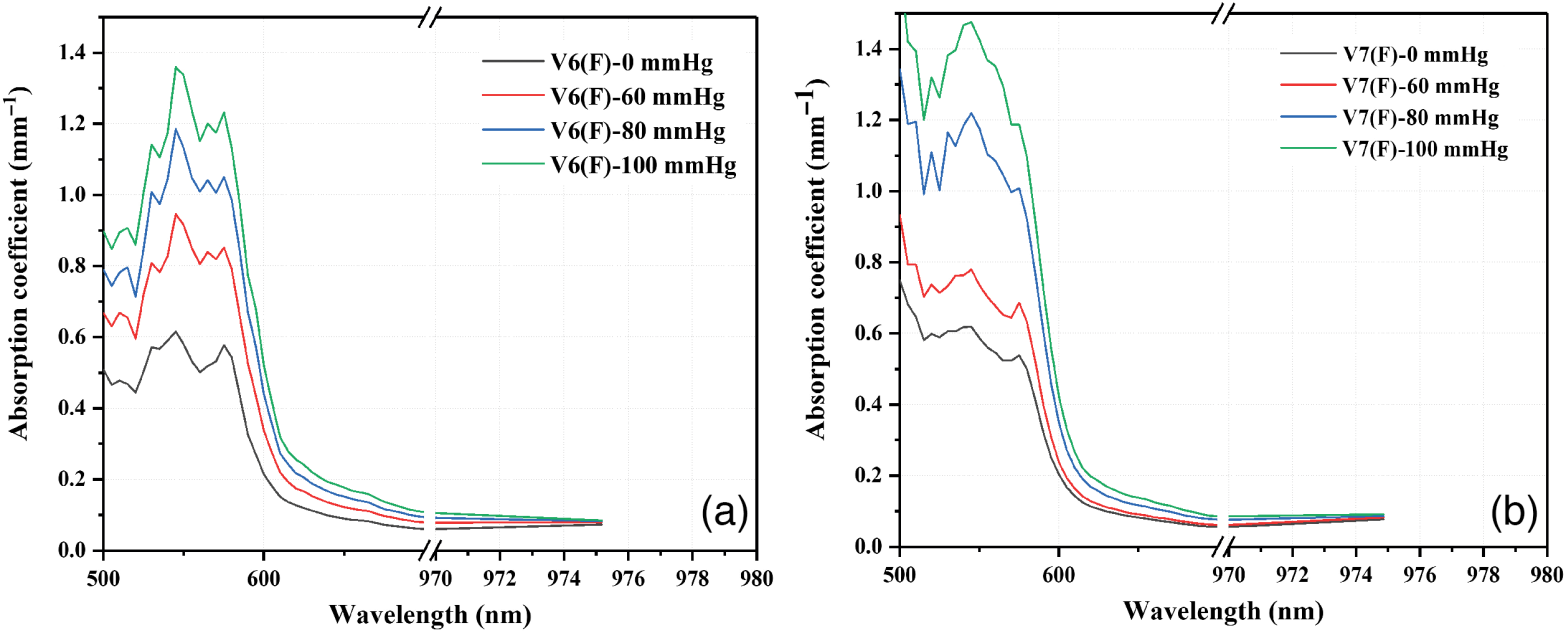
(a) and (b) Absorption coefficients of two volunteers for each condition of applied pressure.

Absorption spectra from diffuse reflectance measurements were obtained using the experimental set-up shown in Fig. 1 and applying the IAD technique. The graphs of Fig. 5 show the absorption coefficients spectra of 2 volunteers for each condition of applied pressure (0, 60, 80, and 100 mmHg) in which it is observed that the absorption coefficient increases with increasing pressure in the sphygmomanometer because of the increase in the concentration of deoxygenated hemoglobin (Hb). Figure 5(a) shows that, by increasing the applied pressure, the characteristic double peak pattern of oxygenated hemoglobin $\left({\mathrm{HbO}}_{2}\right)$ is replaced by the characteristic single peak pattern of deoxygenated hemoglobin (Hb). This behavior is better appreciated in Fig. 5(b) corresponding to a volunteer with a lower systolic pressure. In Fig. 5, the spectra also depend on the skin type because darker skin presents greater absorption.^36^

There are several factors that can affect the measurements, which can be classified as anatomical or physiological; however, a statistical study of experimental data by principal component analysis (PCA) of the main skin chromophores (considering the absorption coefficients of melanin, deoxygenated hemoglobin, oxygenated hemoglobin, water, and fat), revealed that the chromophores that have the greatest effect on the total absorption coefficient are oxygenated and deoxygenated hemoglobin and melanin. PCA details can be found in the Supplementary Material.

### 4.2 *In Vivo* Tissue Oxygen Saturation Measurement with OTOS Algorithm

The ${\mathrm{StO}}_{2}$ measurements were carried out with the developed computational algorithm. The skin type was classified according to three groups: I–fair skin, II–intermediate skin, and III–brown skin.

Subsequently, diffuse reflectance measurements and absorption coefficients were obtained for each pressure condition (0, 60, 80, and 100 mmHg). When obtaining the measurement of 0 mmHg (basal or resting condition), the slope of the absorption spectrum between 630 and 700 nm was calculated because that slope is directly related to the melanin content in the skin.^35^ The slope obtained for each volunteer was used to discriminate the contribution of epidermal melanin to the absorption spectrum. From the value of the slope, the first wavelength necessary to perform the calculation of ${\mathrm{StO}}_{2}$, corresponding to the visible region of the electromagnetic spectrum, was chosen. The second wavelength corresponding to the near infrared region was 975 nm due to the availability of the infrared (IR) laser. The three groups specified for the skin type were related to the obtained slope value because the skin type is directly associated with the melanin content in the skin. Table 1 shows the classification group, as well as the interval of the corresponding slope value and selected wavelength for each group.

**Table 1:** Wavelength determination for ${\mathrm{StO}}_{2}$ calculation (considering the absolute value of the slope).

Group	Skin type	Absolute value of slope 630 to 700 nm $\left({\mathrm{mm}}^{-1}/\mathrm{nm}\right)$	Wavelength (nm).
I	Fair	$\text{Slope}<5\times 10^{-4}$	622
II	Intermediate	$5\times 10^{-4}<\text{slope}<8\times 10^{-4}$	624
III	Brown	$8\times 10^{-4}<\text{slope}$	626

**Fig. 6:**
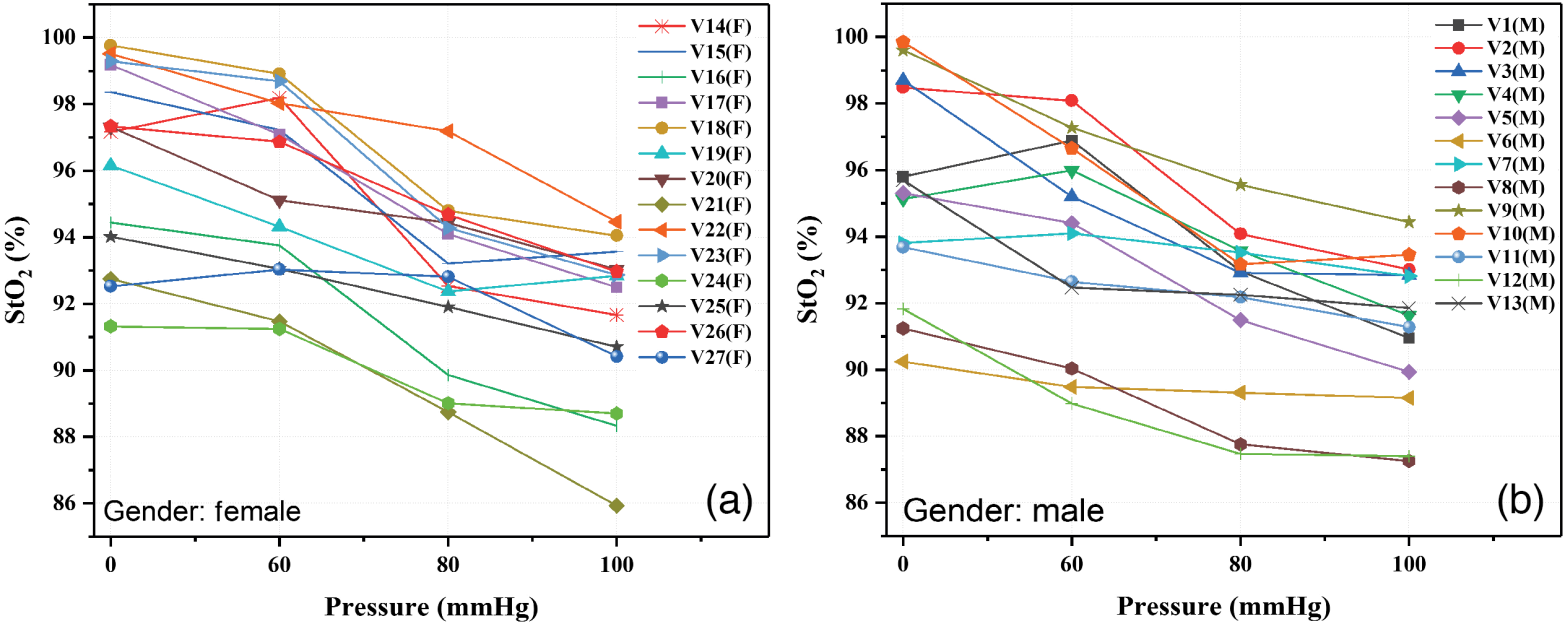
Graphs of ${\mathrm{StO}}_{2}$ values obtained for each pressure condition of occlusion test classified by gender: (a) female and (b) male.

**Fig. 7:**
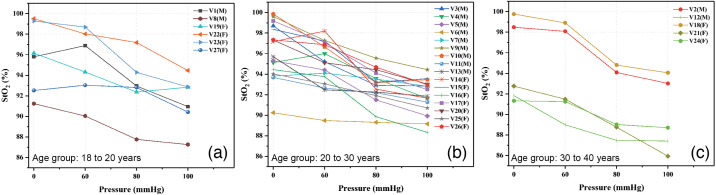
Graphs of ${\mathrm{StO}}_{2}$ for each pressure condition of occlusion test classified by age: (a) 18 to 20 years old, (b) 20 to 30 years old, and (c) 30 to 40 years old.

**Fig. 8:**
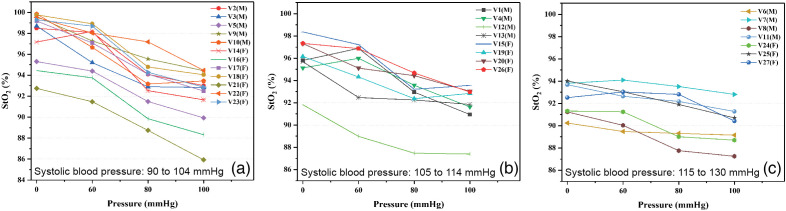
Graphs of ${\mathrm{StO}}_{2}$ values obtained during the occlusion test: systolic blood pressure (a) 90 to 104 mmHg, (b) 105 to 114 mmHg, and (c) 115 to 130 mmHg.

To assign a wavelength for each group of skin type, the behavior indicated by Ferguson-Pell and Hagisawa^37^ was considered. It was observed that there was a change in wavelength in the calculation of the oxygenation index due to the influence of melanin. The obtained values of ${\mathrm{StO}}_{2}$ for each pressure condition during the occlusion test are shown in Figs. 6–8, where it can be seen that the value of ${\mathrm{StO}}_{2}$ tends to decrease as the applied pressure increases. Measurements were categorized in groups by gender, age, and corresponding systolic blood pressure measured before test. Figure 6(a) shows the results belonging to the group of women, and Fig. 6(b) corresponds to the men. Regarding gender, no correlation was found, so it can be concluded that it does not influence the measurement of tissue oxygen saturation.

The classification by age is shown in Fig. 7. The results for the 10 to 20 years old group is observed in Fig. 7(a), for the 20 to 30 years old group in Fig. 7(b), and the 30 to 40 years old group is in Fig. 7(c). No special correlation between volunteers of a similar age was observed. Age influences some physiological characteristics such as hydration and the quantity/structure of collagen fibers present in the skin. However, the volunteers’ ages ranged between 18 and 39 years, which does not represent a considerable difference.

Figure 8(a) shows the results for the group that presented a systolic blood pressure between 90 and 104 mmHg, Fig. 8(b) corresponds to the group with a blood pressure of 105 to 114 mmHg, and Fig. 8(c) shows the group with a blood pressure of 115 to 130 mmHg.

Classifications by gender and age do not present a special pattern or behavior. However, in the classification by group of systolic blood pressure, it is observed that the group with the lowest blood pressure (90 to 104 mmHg) presents a pronounced drop in ${\mathrm{StO}}_{2}$, unlike the group with the highest blood pressure (115 to 130 mmHg). This is an expected behavior because the volunteers with lower systolic blood pressure practically reach a situation of zero blood flow in the highest applied pressure (100 mmHg); therefore they present a greater affectation. It is also expected that, in the absorption spectra of this group of volunteers, the characteristic double peak pattern of oxygenated hemoglobin gets lost faster, as is the case of volunteer V21(M) in Fig. 8(a) which corresponds to volunteer V7(M) in Fig. 5(b).

**Fig. 9:**
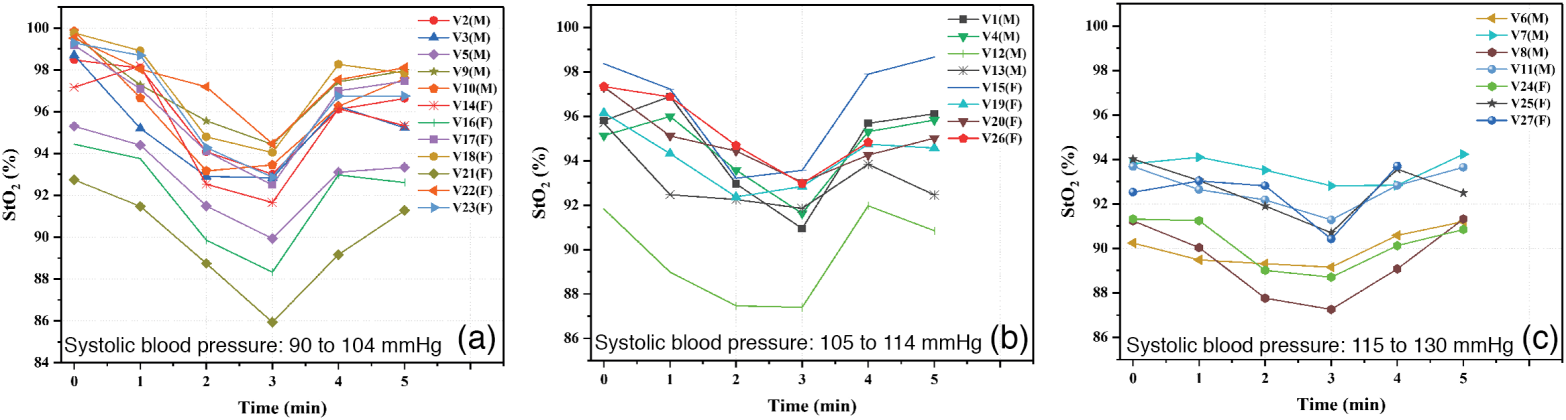
Graphs of ${\mathrm{StO}}_{2}$ values versus test time: volunteer’s initial systolic blood pressure (a) 90 to 104 mmHg, (b) 105 to 114 mmHg, and (c) 115 to 130 mmHg.

Figure 9 shows the graphs of ${\mathrm{StO}}_{2}$ versus time in minutes, where 1 measurement was taken each 1 min. These graphs also show the measurements corresponding to the ${\mathrm{StO}}_{2}$ recovery stage, that is, after evaluating 100 mmHg, the pressure applied by the sphygmomanometer cuff was immediately released and 2 consecutive measurements were taken.

In Fig. 9, it can be seen that the ${\mathrm{StO}}_{2}$ tends to go up again in the recovery stage, and a heterogeneous behavior can be observed between the recovery stage of the different volunteers of each group. Based on observations made in various studies, this recovery stage is considered to be a reflection of the endothelial function and has been correlated with perfusion pressure; therefore, it can be derived from the interaction between perfusion pressure and endothelial integrity.^38^ However, the obtained results indicate a much lower ischemic challenge than that study because the maximum applied pressure (100 mmHg) was not as high as the initial systolic blood pressure and therefore does not present a good degree of comparison. Despite this, in the volunteers V12(H) in Fig. 9(b) and V25(M) in Fig. 9(c) it was observed that, in the first measurement of the recovery stage, the ${\mathrm{StO}}_{2}$ temporarily went up above the initial values. This behavior may indicate the condition known as reactive hyperemia, mentioned in the literature, which represents post-ischemic vasodilation and capillary recruitment.^38^^,^^39^ Although multiple comparisons were made to identify differences between subgroups of volunteers, no statistically significant differences between gender, age, or skin color were found.

The validation of the results obtained in comparison with values from the literature is difficult because there is not a defined standard and previous studies report different results regarding the measurement zones, the separation between the optical probes, and the algorithms used. However, our results showed agreement with those reported by Tseng et al.,^40^ in which they performed diffuse reflectance measurements with visible and near-infrared light and determined two regions to obtain the ${\mathrm{StO}}_{2}$. The region between 500 and 600 nm provide saturation values between 60% and 70% because the depth of penetration is lower and the measurements correspond to the blood vessels of the upper dermis, whereas the region from 600 to 1000 nm provides values of saturation from 97% to 99% by covering a greater depth of measurement in the region of the lower dermis.

The proposed method gives saturation values between 97% and 99% for healthy people under normal conditions; therefore it satisfies the necessary characteristics to be considered a technique for obtaining ${\mathrm{StO}}_{2}$ because it can be applied *in vivo* and non-invasively, requires less evaluation time, and provides an objective and quantifiable evaluation.

As future work, the implementation of the portable device designed to apply the technique in clinical practice will be considered; it will work with LED illumination and photodiodes for detection. In addition, this device has the advantage of changing the size of the port and thus being able to measure smaller areas, allowing for the application of the technique to other possible conditions.

## 5 Conclusions

A technique for measuring tissue oxygen saturation (${\mathrm{StO}}_{2}$) was developed with the aim of monitoring microcirculatory changes in patients at risk of suffering from diabetic foot. The developed technique used an integrating sphere as a measurement device, which reduced the problems of positioning and separation of optical probes; it constituted a portable and flexible measurement method in various applications. The developed procedure measured the diffuse reflectance spectra of the human skin and used the IAD algorithm to obtain the absorption coefficients to obtain the ${\mathrm{StO}}_{2}$ values. The computer program OTOS was developed to automate the implemented methodology and thus facilitate its use in clinical applications.

The developed OTOS program was applied with a vascular occlusion test to assess changes in ${\mathrm{StO}}_{2}$ in a group of 27 volunteers. The occlusion test consisted of placing a sphygmomanometer on the volunteers’ left arms and progressively increasing the pressure to 0, 60, 80, and 100 mmHg while the absorption coefficients in the palm of the left hand were measured. The results showed the expected behavior, according to the information reported in the literature. By increasing the applied pressure, the ${\mathrm{StO}}_{2}$ also decreased by an average of 95.69%, 94.78%, 92.45%, and 91.46%, respectively. It was observed that the volunteers with lower systolic pressures had a greater drop in ${\mathrm{StO}}_{2}$ because the applied pressure of 100 mmHg was above the systolic blood pressure in some of the volunteers. In the measurement of the absorption spectra, it was observed that, by increasing the applied pressure, the characteristic double peak pattern of oxygenated hemoglobin between 500 and 600 nm was replaced by the single peak pattern that characterizes deoxygenated hemoglobin; this behavior indicated that the blood began to deoxygenate.

A statistical study of experimental data by PCA was developed; it was observed that oxygenated and deoxygenated hemoglobin and melanin are the most significant chromophores in the absorption spectrum in the wavelength range under study. However, the developed algorithm is capable of eliminating the effect of melanin by correlating the slope of the absorption spectrum in the interval from 630 to 700 nm with the melanin content; therefore the value of ${\mathrm{StO}}_{2}$ did not depend on the skin type of the person on whom the measurement was made.

From the obtained results, we can conclude that the proposed method meets the necessary characteristics to be considered a technique for obtaining ${\mathrm{StO}}_{2}$ because it can be applied *in vivo* and non-invasively and does not require a high computational cost; thus it is fast and capable of providing an objective and quantifiable evaluation. For patients at risk of developing diabetic foot, the proposed method can help quantify the decrease in ${\mathrm{StO}}_{2}$ as an indication of the reduction in limb blood flow during follow-up medical visits.^41^^–^^48^

## Code, Data, and Materials Availability

Code and data will be made available on request.
