# Supplementary material for: Determination of tissue oxygen saturation by diffuse reflectance spectroscopy
Source: J Biomed Opt. 2023 Sep 28;28(9):095002. doi: 10.1117/1.JBO.28.9.095002 (PMC10534074; doi:10.1117/1.JBO.28.9.095002)
Supplement: Supplementary file 1 [file JBO_028_095002_SD001.pdf]

Supplementary material: Determination of tissue oxygen saturation by diffuse reflectance spectroscopy

I. Classification of volunteers according to their skin color

According to Karsten and Smit [27] the absorption coefficient of human skin is proportional to the melanin content, that is, its skin type.

A diffuse reflection spectrum was measured on the palm of the hand for each volunteer and its absorption spectrum was calculated, following the methodology described by Quistián [23]. In figure S1, the wavelength of 632 nm was chosen to show a comparison between the different skin types. The skin type label was obtained from a questionnaire based on the Fitzpatrick criteria.

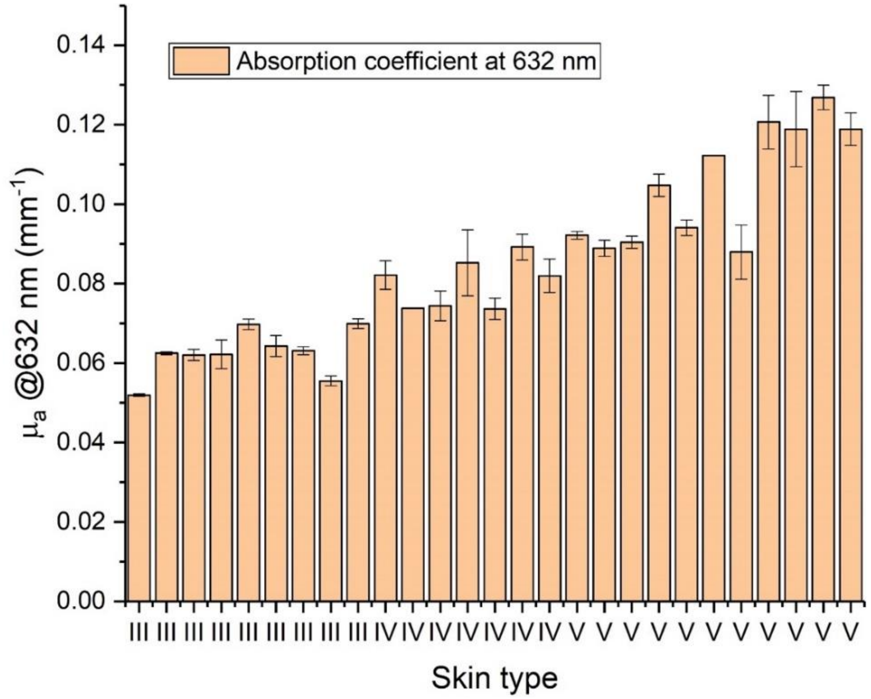

Figure S1. Experimental absorptioncoefficient of the volunteers.

II. Statistical analysis of the volunteers number

To perform the experimental tests, it is necessary to consider an appropriate sample size, since considering a very large sample size may imply a waste of resources and a very small size may lack practical use [41]. Therefore, the number of volunteers(n) was obtained from equation S1, which indicates a relationship between the standard deviation (σ), reliability (z) and the desired confidence interval dimension (d):

$$n = \frac{z^2\sigma^2}{d^2} \qquad (S1)$$

where z depends on the confidence level $(1-\alpha)$ and α represents the significance level. For example, for a value of $\alpha = 0.01$ the confidence level is equal to 99% and its reliability will be $z = 2.58$, where z is obtained by matching the value of the confidence interval to $erf\left(z/\sqrt{2}\right)$ with erf the

Gaussian error function. The value of σ can be obtained from a preliminary sample (n_1) where a fitting is subsequently applied to the calculated sample size (n) and a number of observations needed to complete the total sample size requirement is obtained.(n_2), as indicated by equation S2:

$$n - n_1 = n_2 \qquad (S2)$$

In this work, a confidence level of 95% was considered, which provides a value of $z = 1.96$, a confidence interval dimension $d = 1$ and a standard deviation $\sigma = 2.65$ (obtained from a preliminary sample of 64 measurements with 16 volunteers as shown in table S1). Therefore, substituting these values in equation S1, a sample size is obtained: $n = 26.97 \approx 27$ volunteers.

	StO_2 (%)			
Volunteer	0 mmHg	60 mmHg	80 mmHg	100 mmHg
V1	89.59	90.03	87.76	87.26
V2	97.33	96.87	94.68	92.97
V3	92.53	93.03	92.82	90.42
V4	93.06	94.10	93.51	92.81
V5	90.24	89.48	90.58	89.31
V6	98.48	98.09	94.08	93.01
V7	98.69	95.20	92.91	92.84
V8	99.76	98.91	94.80	94.05
V9	94.44	93.75	89.86	88.33
V10	94.94	95.99	93.58	91.62
V11	97.17	98.19	92.53	91.66
V12	92.38	91.47	88.74	85.93
V13	96.16	94.31	92.37	92.84
V14	95.80	96.89	92.96	90.95
V15	98.36	97.22	93.21	93.56
V16	99.18	97.08	94.10	92.49
Standard deviation	3.205	2.905	2.088	2.392

Table S1. Preliminary sample standard deviation for sample size calculation. The mean of the standard deviation is 2.65.

The volunteers number obtained was compared with other studies that also applied a vascular occlusion test to healthy volunteers. Table S2 shows the review of the volunteers number that were used in mentioned studies, where it can be seen that the number of volunteers that we consider in this work is within the reported range.

Author	Year	Volunteers number
Abay et al. [42]	2016	19 healthy volunteers
Donati et al. [25]	2016	27 healthy volunteers
Hartwig et al. [43]	2016	12 healthy volunteers
Boezeman et al. [44]	2015	8 healthy volunteers
Fellahi et al. [45]	2014	20 healthy volunteers
Bezemer et al. [26]	2009	8 healthy volunteers

Table S2. Review of volunteers number in various studies.

III. Principal Components Analysis

There are different chromophores present in the human skin (hemoglobin, melanin, water, etc.) these exhibit a characteristic absorption spectrum. Principal component analysis (PCA) can be applied to linearly transform data from an orthogonal coordinate system. This procedure indicates that the principal components corresponding to the axes of the system are determined as eigenvectors of the covariance matrix of the data set, and their corresponding eigenvalues refer to the variance captured within each eigenvector [46].

A PCA on the main chromophores of the skin was carried out (considering their respective absorption coefficients), degree of correlation between skin chromophores was evaluated, as can be seen from the table S3. The molar concentration of hemoglobin of 64,500 gr/mol was used and for the coefficients of the rest of the skin chromophores, a specific concentration was not used.

	μ_a Hb	μ_a HbO_2	μ_a Melanin	μ_a Water	μ_a Fat
μ_a Hb	1	0.93261	0.73298	-0.22407	0.38804
μ_a HbO_2	0.93261	1	0.68443	-0.173	0.40171
μ_a Melanin	0.73298	0.68443	1	-0.42775	0.70972
μ_a Water	-0.22407	-0.173	-0.42775	1	0.24263
μ_a Fat	0.38804	0.40171	0.70972	0.24263	1

Table S3. Correlation matrix between main skin chromophores.

The data used for the PCA were obtained from the references mentioned on Jacques & Prahl omlc page and the units were converted to mm^{-1}. The absorption coefficients of fat [47] and hemoglobin [29] were taken directly. For the water absorption coefficient [48], a 4th order polynomial fit was performed to identify the values between the wavelengths of interest (500-700 nm and 974 nm), while for the melanin absorption coefficient [31] were obtained from the fit equations S3 and S4, considering the power factor (*m*=3) and the melanosome volume fraction (f_v =0.87).

$$\mu_{a,melanosome} = (519\ \mathrm{cm}^{-1})\left(\frac{\lambda}{500\ \mathrm{nm}}\right)^{-m}, \tag{S3}$$

$$\mu_a due\ to\ melanin\ =\ f_{v,melanosome}\ \mu_{a,melanosome}. \tag{S4}$$

The percentage variance corresponding to the eigenvalues was also evaluated, as it is shown in the table S4, as well as the matrix of the eigenvectors generated in the analysis (table S5).

	Eigenvalue	***Percent variance***	***Cumulative***
1	3.00996	60.20%	60.20%
2	1.22078	24.42%	84.61%
3	0.68629	13.73%	98.34%
4	0.06989	1.40%	99.74%
5	0.01308	0.26%	100%

Table S4. Correlation matrix between eigenvalues.

	PC1 coefficients	*PC2 coefficients*	*PC3 coefficients*
μ_a Hb	0.52632	-0.07179	0.43301
μ_a HbO_2	0.51622	-0.02714	0.48701
μ_a Melanin	0.53618	-0.04384	-0.42394
μ_a Water	-0.17215	0.81242	0.38467
μ_a Fat	0.37333	0.57633	-0.49763

Table S5. Eigenvector matrix.

The graphs of figure S2 show that the chromophores with the greatest effect on the total absorption coefficient are oxygenated hemoglobin, deoxygenated hemoglobin, and melanin. Figure S2b shows that these three chromophores with the greatest contribution are also more related to each other, since these present a small angle between their eigenvectors.

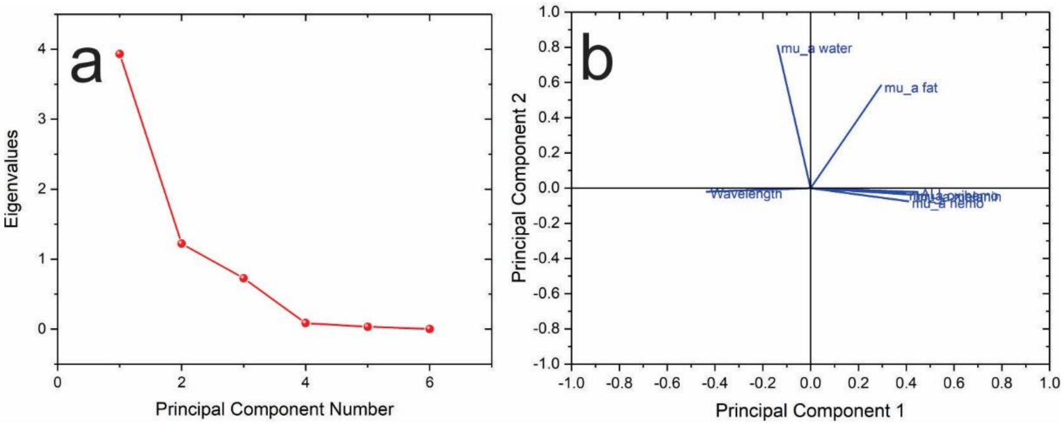

Figure S2. Graphs obtained from principal components analysis: a) Scree Plot, and b) Loading Plot.
